# The “Pacific Coast Dentist”

**Published:** 1893-08

**Authors:** 


### THE "PACIFIC COAST DENTIST."

We have received the first number (June) of this journal. It is under the charge of Joseph D. Hodgen, D.D.S., assisted by a number of dentists in San Francisco and elsewhere on the coast.

The initial number gives promise of a permanent place in the literature of that section of the country, the contents being above the average of first numbers.

The name does not seem to be happily chosen, it not being adapted, in our opinion, to a monthly professional journal. We wish our contemporary a successful career in the journalistic field.

